# The nature of the annular ligament: a scoping review and histological analysis using a dual approach

**DOI:** 10.1016/j.jseint.2025.07.002

**Published:** 2025-08-05

**Authors:** Floor A.J. ten Have, Steef H. Boekholt, Niels W. Schep, Jeffrey Damman, Gert J. Kleinrensink

**Affiliations:** a Department of Neuroscience and Anatomy, Erasmus University Medical Center, Rotterdam, The Netherlands; b Department of Trauma Surgery, Maasstad Hospital, Rotterdam, The Netherlands; c Department of Pathology, Erasmus University Medical Center, Rotterdam, The Netherlands

**Keywords:** Annular ligament, Radial head, Histology, Tissue, Fibrocartilage, Wrap-around ligament

## Abstract

**Background:**

The annular ligament (ANL) is a crucial structure of the elbow joint, playing a vital role in maintaining stability and function of the radial head (RH). Despite its known anatomical and stabilizing roles, the histological composition of the ANL remains unclear. The first article to describe its nature introduced the concept of a wrap-around ligament. A wrap-around ligament is defined as “any tendon that bends around a bony pulley or threads through a fibrous 1 en route to its insertion”. These ligaments are often fibrocartilaginous due to an adaptation to compressive forces. During pronation and supination the ANL is compressed against the RH. Therefore, the authors hypothesize that the ANL contains (fibro)cartilaginous tissue where the ligament is compressed against and experiences friction from the RH. Understanding the precise histological nature of the ANL is essential for surgical procedures involving the RH, such as fracture osteosynthesis, in which hardware placement may lead to complications.

**Methods:**

This study consisted of 2 components: a scoping review and a histological analysis. First, a scoping review was conducted in accordance with the Preferred Reporting Items for Systematic Reviews and Meta-Analyses Extension for Scoping Reviews. A systematic literature search was performed using Embase, Medline, Web of Science Core Collection, Google Scholar, and Cochrane Library. Published literature was screened, and articles were included if they addressed the ANL and its histology or tissue composition. In addition to the review, a histological analysis was performed on 3 ANLs from postmortem human specimens.

**Results:**

A total of 2,453 articles were identified, of which 7 met the eligibility criteria. The literature search revealed discrepancies regarding the presence and location of fibrocartilage and synovial linings within the ANL. Histological examination of 3 postmortem human specimens samples showed (fibro)cartilage cells in 1 ligament and chondrification in another. These features were located in the central part of the ANL, where it articulates with and is compressed against the RH. The third ligament showed no fibrocartilage cells. All 3 ANLs exhibited a synovial lining on the lateral side where the ligament attached to the ulna, but not in the center part of the ligament, between the articulating surfaces of the ANL and the RH.

**Conclusion:**

The ANL is a wrap-around ligament and contains (fibro)cartilaginous tissue. There was no synovial lining present on the articulating surface of the ANL between the ANL and the RH.

The annular ligament (ANL), encircling the proximal radial head (RH),^2^ provides functional stability to the elbow.^10^ It represents a thickening of the joint capsule of the proximal radio-ulnar joint^2^ and keeps the RH in place.^11^ The ANL primarily contributes to lateral stability of the elbow and is therefore considered part of the lateral ligament complex.^26^^,^^31^ It functions in conjunction with the radial collateral ligament and the lateral ulnar collateral ligament, both of which are positioned superficially to the ANL.

The ANL attaches to the anterior and posterior margins of the radial notch of the ulna. Posteriorly, the ANL attaches to the ulna as several bands, that is, the ANL proper and the superior and inferior oblique bands of the ANL (Fig. 1). Anteriorly, the ANL attaches as a single unit to the radial notch of the ulna.^3^^,^^6^^,^^19^

**Figure 1:**
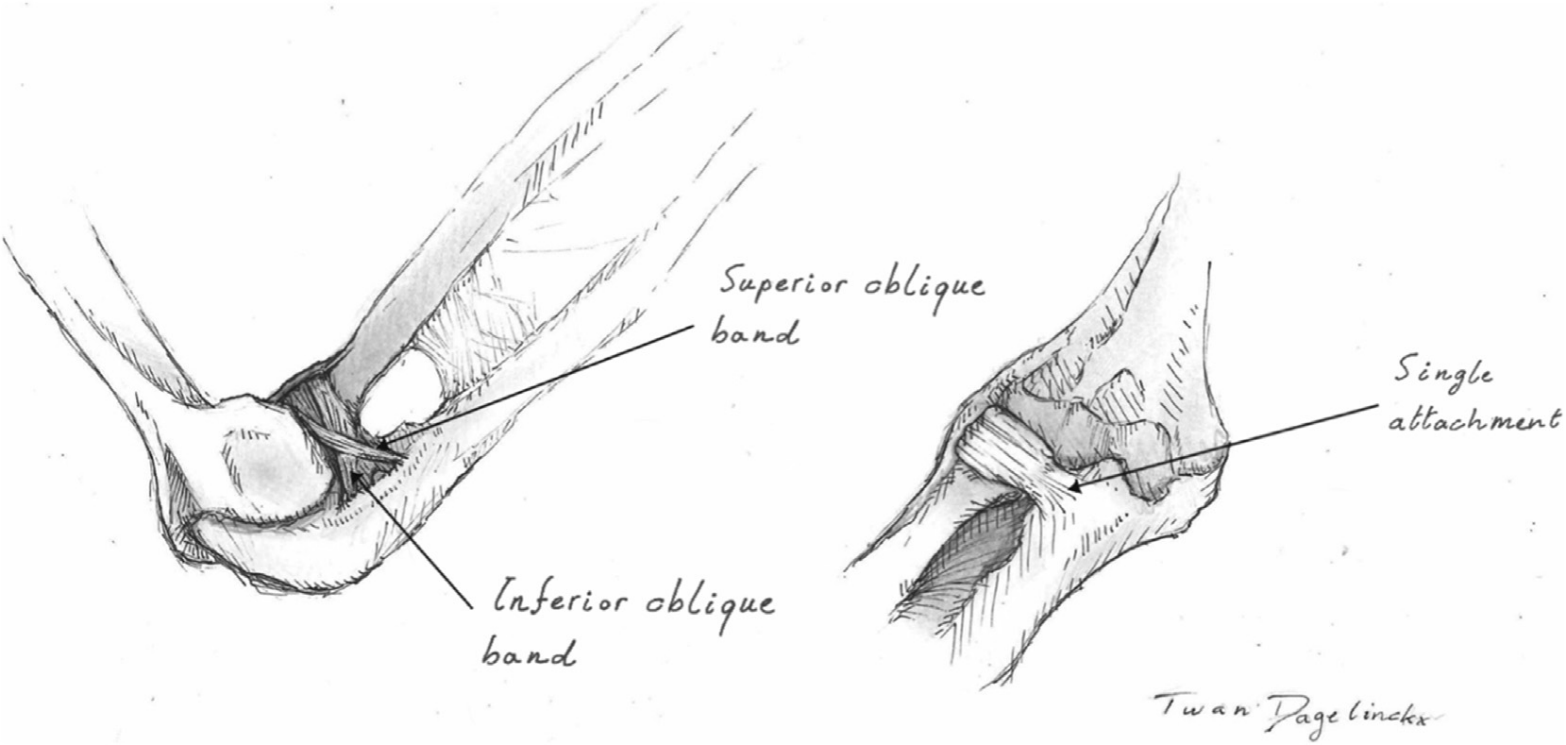
Drawing of the annular ligament. The annular ligament encircling the radial head (*Left*) and the annular ligament and the annular ligament with its superior and inferior oblique bands (*Right*). Drawings made by T Dagelinckx.

Although its anatomy and restraining role in dislocations of the proximal radioulnar joint are widely acknowledged in the medical profession,^2^^,^^3^^,^^6^^,^^7^^,^^11^^,^^23^^,^^26^^,^^31^ few articles have described the histology of the ANL. The first article defining the nature of the ANL^5^ used the term wrap-around ligament. A wrap-around ligament is “any tendon that bends around a bony pulley or threads through a fibrous 1 en route to its insertion”. Wrap-around ligaments are frequently fibrocartilaginous due to adaptations to compressive forces. Ligaments physiologically adapt their molecular composition in accordance with the applied mechanical forces.^16^ This is known as Wolff's law.^7^ A clear example of a wrap-around ligament is the transverse ligament of the atlas. Fibrocartilaginous tissue is present where the ligament is pressed against the dens axis.^18^ Similarly, during pronation and supination the ANL is compressed against the RH. Ergo, the authors hypothesize that the ANL contains (fibro)cartilaginous tissue where the ligament is compressed against and experiences friction from the RH. Supporting this theory, Gray's Anatomy states that the internal surface of the ANL has a thin coating of cartilage.^8^

Understanding the nature of the ANL is essential for performing surgical interventions in this region. Surgical treatment options for RH fractures depend on fracture type and include fragment excision, open reduction internal fixation (ORIF), RH excision, and prosthetic arthroplasty.^22^ During ORIF procedures, osteosynthetic hardware is frequently inserted between the RH and ANL.^20^ It is conceivable that the complications associated with placing osteosynthesis materials within a joint cavity may also apply to wrap-around ligaments. How does a wrap-around ligament, which closely articulates with the surface of a bone, differ from a true joint in terms of function and movement? In clinical practice, many patients experience pain due to osteosynthetic material inserted between the RH and ANL, necessitating its subsequent removal.^32^ Therefore, precise characterization of the ANL is of clinical importance. This study aims to comprehensively review the existing literature on the histology and tissue composition of the ANL to identify knowledge gaps through a scoping review. In addition, histological examinations of several ANLs from postmortem human specimens (PMHS) were conducted to accurately define the nature of the ANL.

## Materials and methods

### Primary outcome

The aim of this review is to accurately define the nature of the ANL. To achieve this, the study comprised 2 components: a scoping review and an original histological analysis of ANLs dissected from embalmed PMHS. The scoping review systematically summarized all available literature on the tissue composition, histology, or nature of the ANL. To address discrepancies identified in the literature, a histological examination was subsequently performed on 3 dissected ANL specimens from embalmed PMHS, with the aim of clarifying the histological nature of the ANL.

### Search strategy

A literature search was conducted using multiple databases, namely Embase, Medline, Web of Science Core Collection, Cochrane, and Google Scholar on November 19, 2024. The search strategy was developed by a library scientist of the medical library of the Erasmus Medical Center (Table I). The scoping review was based on the Preferred Reporting Items for Systematic Reviews and Meta-Analyses Extension for Scoping Reviews.^27^

**Table I:** Search strategy.

Database searched	Search strategy∗
Embase	('annular ligament'/de OR ('radial head'/de AND ('ligament'/de)) OR (((radial OR radius) NEAR/3 head∗ NEAR/6 ligament∗) OR (annular∗ NEAR/3 ligament∗)):ab,ti)
Medline ALL	((((radial OR radius) ADJ3 head∗ ADJ6 ligament∗) OR (annular∗ ADJ3 ligament∗)).ab,ti.)
Web of Science Core Collection	TS=(((((radial OR radius) NEAR/2 head∗ NEAR/5 ligament∗) OR (annular∗ NEAR/2 ligament∗))))
Cochrane Central Register of Controlled Trials	((((radial OR radius) NEAR/3 head∗ NEAR/6 ligament∗) OR (annular∗ NEAR/3 ligament∗)):ab,ti) ("conference abstract":kw OR Trial registry record:pt) #! NOT #2
Google Scholar	'radial|radius head ligament|ligaments'|'annular ligament|ligaments '

∗ The search strategy was designed by a library scientist of the medical library of the Erasmus Medical Center (Erasmus MC), dr. W. Bramer.

### Eligibility criteria and inclusion

Articles were included if they provided information on the histology or tissue composition of the ANL of human elbows. Only articles written in English were included. To minimize publication bias, no restrictions were applied regarding publication date. Articles focusing on traumatic history of the elbow as well as secondary sources, such as reviews and guideline documents, were excluded. The reference lists of all included articles were screened for eligibility. Corresponding authors were contacted twice if an article was unavailable. The authors (SB and FtH) independently selected articles identified through the literature search based on their titles and abstracts. All selected articles were secondarily assessed on full text. Any discrepancies were resolved by discussion between the 2 authors (SB and FtH) and their supervisors (NS and G-JK).

### Data extraction and synthesis

Data were extracted from all eligible articles by each author independently (SB and FtH). For each article, information was collected on the author, publication date, country of origin, number of elbows studied, and primary outcome. The articles were summarized and categorized in the following categories: “Embryology and the supinator muscle”, “Histology and synovial lining” and “Strata”.

### Quality assessment

All included articles were evaluated using an original scoring system (Table II). None of the JBI critical appraisal tools^13^ were applicable for the expected eligible articles. Therefore, the authors developed a scoring system based on the different JBI critical appraisal tools. The quality was assessed using the following categories: Number of included cases/arms, representativeness of cases/potential of selection bias, clarity of definition of primary end point, clarification of histological methodology, depth of observation detail, analytic depth, and validity. In the quality assessment, the clarification of the histological process and the representativeness of the cases were prioritized over other categories, as the authors found these categories to be of the greatest importance. Cases were deemed representative if the authors selected the arms randomly and if the baseline characteristics of the cases were well described. All included articles were independently assessed by the 2 authors. If there were any discrepancies, these were resolved by discussion between the 2 first authors (SB and FtH), aided by one of the other authors (NS and G-JK). The authors deemed an article excellent with a score of 21-24 points, good 17-20 points, medium 13-16 points, low 7-12, and poor 0-6 points. Notably, as this was a scoping review aiming to map the literature and indicate the knowledge gaps, all articles meeting the eligibility criteria were included, regardless of quality score.

**Table II:** Quality assessment scoring system

Quality category	Points
Number of cases/arms	
1-9 arms	0
10-19 arms	1
20-29 arms	2
30-39 arms	3
40-49 arms	4
≥50 arms	5
Representativeness of cases	
No description of baseline characteristics arms	0
Arms were randomly chosen	2
Description of baseline characteristics arms given	2
Description of baseline characteristics arms given and arms were randomly chosen	4
Clarity of definition of primary end point	
Primary end point not defined	0
Primary end point inadequately defined	1
Primary end point defined well	2
Clarification of histological method	
Clarification of examined part of ligament	
No clarification	0
Medium clarification	2
Detailed clarification	4
Clarification of histological process	
No clarification	0
Medium clarification	2
Detailed clarification	4
Depth of observation detail results	
Medium depth	0
Detailed depth	2
Analytic depth	
No analytic depth	0
Analytic depth	1
Validity	
Conclusion does not correspond with the primary end point	0
Conclusion inadequately corresponds with the primary end point	1
Conclusion does correspond with the primary end point	2
Total maximum points	24

### Histological examination

The ANL was harvested from 3 different PMHS. The specimens were obtained from body donors who had given written informed consent and were part of the Dutch national donor program, which complies with Dutch legal requirements. The PMHS were fixed with AnubiFix^24^ and 10% formalin solution for more than three months. Elbows were excluded from examination if there were signs of previous surgical intervention in or around the elbow joint. Dissection was performed by an experienced senior anatomist. The skin and underlying functional structures, such as muscles, nerves, and vessels were removed, leaving the elbow joint capsule intact. The joint capsule was then opened to expose and finally dissect the ANL. The ANL was severed at the anterior and posterior margin of the radial notch at the ulna. All dissected structures were marked with sutures, providing orientation for processing and microscopy. 4 μm-thick paraffin-embedded sections of the ANL were prepared at intervals of 10−20 (100−200) μm. The ANL was sectioned sagittally and stained with hematoxylin and eosin, with additional staining using Alcian blue. Both staining techniques were used for evaluation under light microscopy.

Histological interpretation was performed by an experienced pathologist (JD) and focused on the presence of (fibro)cartilaginous tissue and synovial lining, particularly in areas where the ligament was compressed against and experienced friction from the RH.

## Results

### Study selection

**Figure 2:**
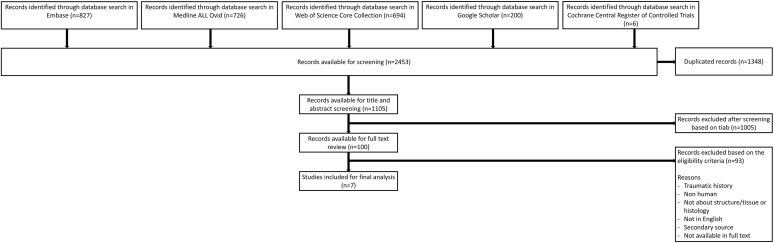
Flowchart of study selection using the eligibility criteria.

A total of 2,453 articles were identified using the aforementioned search strategy (Table III). After removal of duplicates, 1,105 articles remained and were screened based on title and abstract according to the eligibility criteria. Following this screening, 100 articles were selected for full-text review. Reference checking did not result in additional relevant records. Ultimately, 7 articles describing the tissue composition or histology of the ANL were included in this scoping review (Fig. 2).

**Table III:** Results search strategy. No other database limits were used than those specified in the search strategies.

Database searched	Platform	Yr of coverage	Records	Records after duplicates removed
Medline ALL	Ovid	1946-Present	726	723
Embase	Embase.com	1971-Present	827	188
Web of Science Core Collection∗	Web of Knowledge	1975-Present	694	139
Cochrane Central Register of Controlled Trials	Wiley	1992-Present	6	1
Additional Search Engines: Google Scholar†			200	57
Total			**2,453**	**1,108**

∗ Science Citation Index Expanded (1975-present); Social Sciences Citation Index (1975-present); Arts and Humanities Citation Index (1975-present); Conference Proceedings Citation Index- Science (1990-present); Conference Proceedings Citation Index- Social Science and Humanities (1990-present); Emerging Sources Citation Index (2005-present).

† Google Scholar was searched via "Publish or Perish" to download the results in EndNote.

#### Article characteristics

The 7 included articles comprised a total of 263 elbows ranging from 6 to 72 elbows per article. The studies were conducted in Japan, the United States of America, Germany, India, and the United Kingdom between 1958 and 2022. An overview of the characteristics of the included articles is provided in Table IV.

**Table IV:** Baseline Characteristics. An overview of the characteristics of the articles selected.

Author (publication date)	Country	Number of elbows	Age at death (yr)	Gender	Primary outcome
Anand et al^1^	India	72	Unknown	Both sexes	The aim of the study was to ascertain the nature of the cartilage of the ANL microscopically as well as look for any variations and macroscopic changes which could provide a clue for indeterminate causes for pain around the elbow for an orthopaedician.
Bartz et al^4^	Germany	60	29-99	32 female, 28 male	This article presents a contribution to the study of the biomechanics of the proximal radio-ulnar joint. In morphological and photoelastic experiments, the importance of the annular ligament for the transmission of force is investigated. The distribution of the osseous material in the subchondral bone and the structure of the cancellous bone are examined in photoelastic model tests with respect to the degree and type of load applied.
Hayashi et al^12^	Japan	50	10−32 weeks of gestation	Unknown	The aim of this study was to clarify the development of the supinator muscle and ANL and coccygeus muscle and sacrospinous ligament in human fetuses.
Martin et al^18^	United kingdom	50	Unknown	Unknown	This study has shown that the superior radio-ulnar joint has the same fundamental structure as other synovial joints, in so far as it possesses a capsule, which is lined by synovial lining, and reinforced externally by accessory ligaments.
Sanal et al^25^	United states of america	6	Mean of 80.7 (range, 64-87)	2 female, 4 male	The purpose of this study is to provide a detailed analysis of the anatomy of the ANL using MR arthrography with anatomic and histologic correlation in cadavers'.
Tsuji et al^31^	Japan	14	Median of 79 (range, 75-88)	Unknown	The objective of this study was to clarify the arthroscopic, macroscopic, and microscopic anatomy of the radiocapitellar synovial fold of the elbow joint in correlation with the common extensor origin.
Lühmann et al^16^	Germany	11	Median of 72 (range, 67-75)	6 female, 2 male	The aim of this study was to compare the histomorphology of the elbow capsule and its ligaments to gain a better understanding of the clinically relevant biomechanical stabilization.

#### Quality assessment

Using the original scoring system, none of the articles received an excellent or poor score. Three articles were of good quality, 2 articles scored a medium score, and 2 articles scored a low score (Table V).

**Table V:** Quality assessment results.

	Anand et al^1^	Bartz et al^4^	Hayashi et al^12^	Martin et al^18^	Sanal et al^25^	Tsuji et al^31^	Lühmann et al^16^
Number of cases/arms	5	5	5	5	0	1	1
Representativeness of cases							
Arms were randomly chosen	2	2	0	2	0	0	0
Description of baseline characteristics arms given	0	2	0	0	2	2	2
Clarity of definition of primary end point	2	1	2	1	2	2	2
Clarification of histological method							
Clarification of examined part of ligament	0	2	4	4	2	2	4
Clarification of histological process	2	2	4	0	4	2	4
Depth of observation detail results	0	0	2	0	0	0	2
Analytic depth	0	1	1	0	1	0	1
Validity	2	2	2	0	2	2	2
Total allocated points	13	17	20	12	13	11	18
Category	Medium	Good	Good	Low	Medium	Low	Good

### Histology of the ANL in literature

Six of the 7 articles performed a histological examination (Table VI). Anand et al.^1^ showed that the ANL consisted of white fibrocartilage. Dense fibrous tissue was arranged in bundles interspersed with fibroblasts and small chondrocytes arranged in groups. However, they did not specify the location of the white fibrocartilage within the ligament. Furthermore, they observed pitting similar to that of bony articulating surfaces. Bartz et al.^4^ described 2 zones of different histological composition within the ANL. The 2 outer parts of the ANL formed 1 zone, the center or middle part (the area opposite the radial notch of the ulna) formed the other zone. In the center part toward the articular cavity, bundles of collagen fibers mostly ran parallel to the RH. On the outer side, the fibers crossed each other at various angles, the tissue was made up of firm fibrous material and only fibroblasts were found. However, in the center part of the ANL, cartilage cells were found either individually or in clusters alongside fibroblasts. Here, the collagen fiber bundles created an 'elongated latticework.' Bartz et al.^4^ also performed staining with Alcian blue (pH 1.0). This led to concentrated blue staining in the inner third of the ANL, less intensive staining in the middle third, and no staining on the outer side of the ANL. The reaction was weaker at the transition to the anterior and posterior parts of the ANL and eventually disappeared. In summary, the outer parts contained only fibroblasts, while in the center cartilage cells were evident on the inner or articulating side of the ligament.

**Table VI:** Summary of the histological findings in literature.

Article	Histology of the ANL	Synovial lining on the articulating side of the ANL	Strata of the ANL
Anand et al^1^	White fibrocartilage Dense fibrous tissue Chondrocytes in groups	No description of the presence of synovial lining	No description of strata
Bartz et al^4^	Center part: bundles of collagen fibers, cartilage cells Outer part: fibroblasts Dorsal and palmar regions: firm fibrous tissue	No description of the presence of synovial lining	No description of strata
Hayashi et al^12^	No description of the presence of cartilage cells or cartilaginous tissue	No description of the presence of synovial lining	No description of strata
Sanal et al^25^	No description of the presence of cartilage cells or cartilaginous tissue	A thickening of the joint capsule around the radial head with a thin synovial lining that was continuous superiorly and inferiorly with the elbow joint Capsule	Innermost stratum: synovial lining Middle stratum: thickened part of the capsule (i.e. The annular band) Outermost stratum: contributions from the lateral collateral ligamentous complex and the supinator muscle
Tsuij et al^31^	No description of the presence of cartilage cells or cartilaginous tissue	Synovial lining on the innermost or articulating part	Innermost stratum: layer of synovial lining Intermediate stratum: the actual ANL Third stratum: dense irregular fibrous connective tissue Outermost stratum: tendon of the common extensor origin
Lühmann et al^16^	Different types of collagenous fiber arrangements	No description of the presence of synovial lining	No description of strata
Martin et al^18^	Did not perform histological examination	Synovial lining distal or lowermost part of ANL attached to the neck of the radius	Innermost stratum: continuous with and formed by the capsule of the joint Intermediate stratum: the annular band Outermost stratum: derived from the collateral ligament

In contrast to these findings, 4 articles^12^^,^^15^^,^^23^^,^^29^ performed a histological examination of the ANL but did not report the presence of cartilage cells or cartilaginous tissue in the ANL. Martin et al.^17^ did not perform any histological examination of the ANL.

Lastly, Lühmann et al.^15^ described different types of collagenous fiber arrangements in the elbow joint capsule. Using polarization microscopy, they classified the anisotropic arrangement of collagen fibers into 4 types: densely packed parallel, mixed tight and loose parallel, densely interlaced, and mixed tight and loose interlaced. They observed that the ANL consisted of a few densely packed interlaced fibers, and a few mixed tight and loose parallel fibers, but mostly consisted of densely packed parallel fibers. They concluded that the densely packed parallel fiber arrangements reflect the stabilizing function of the ANL.

### Synovial lining on the articulating side of the ANL

Sanal et al.^23^ reported that the ANL was continuous with the joint capsule and could be considered as a thickening of the joint capsule surrounding the RH. They observed a synovial lining on the innermost part of the ANL, where it was continuous with the joint capsule. These findings were supported by Tsjui et al.,^29^ who stated that the innermost stratum of the ANL consisted of a layer of synovial lining. Both studies therefore suggest the presence of a synovial lining on the articulating surface of the ANL, that is, between the ANL and the RH. In contrast, Martin et al.^17^ reported that the synovial lining was only present in the distal or lowermost part of the ANL under a thin fibrous covering. According to their observations, the synovial lining was present at the point where the ANL attached to the neck of the radius but not on the articulating surface between the ANL and the RH. The presence of a synovial lining was not described in the remaining 4 included articles.^1^^,^^4^^,^^12^^,^^15^

### Strata of the ANL

Tsuji et al.,^29^ Martin et al.^17^ and Sanal et al.^23^ concluded that the ligament is composed of different strata. According to Tsuji et al.,^29^ the innermost stratum comprised a layer of synovial lining, while the intermediate stratum represented the actual ANL; the third stratum comprised dense irregular fibrous connective tissue, and the outermost stratum was formed by the tendon of the common extensor origin. Similarly, Sanal et al.^23^ described an innermost stratum that consisted of a synovial lining, followed by a thickened part of the capsule (ie the annular band), and lastly, contributions from the lateral collateral ligamentous complex and the supinator muscle formed the outermost stratum. However, Martin et al.^17^ concluded that the ligament consisted of 3 strata. They described an innermost stratum which is continuous with and formed by the capsule of the joint. The intermediate stratum consisted of the annular band and the outermost stratum was derived from the collateral ligament. Furthermore, Martin et al.^17^ noted that the ANL could be divided into an anterior and posterior part. Posteriorly, the 3 strata were distinguishable, whereas anteriorly the 2 innermost strata were integrated. The authors observed that posteriorly a probe was able to pass between the annular band and ‘deep band’ or innermost stratum of the ligament. However, more anteriorly, where the strata fuse, this was no longer possible.

### Embryology and the supinator muscle

Hayashi et al.^12^ described the influence of developing ligaments in fetuses and the muscles attached to them. At week 10, the ANL composed of loose fibrous tissue; the fibers of the primitive ANL were continuous with the tendon of the supinator muscle. At week 18, the supinator tendon was tightly attached to the ANL, making it difficult to distinguish the tendon from the ligament. At week 22, both the ANL and supinator muscle were well developed. The thick and tight ANL contained several long muscle fibers originating from the supinator muscle. In conclusion, Hayashi et al.^12^ demonstrated that the developing ANL incorporated muscle fibers from the supinator muscle.

### Histological examination

**Figure 3:**
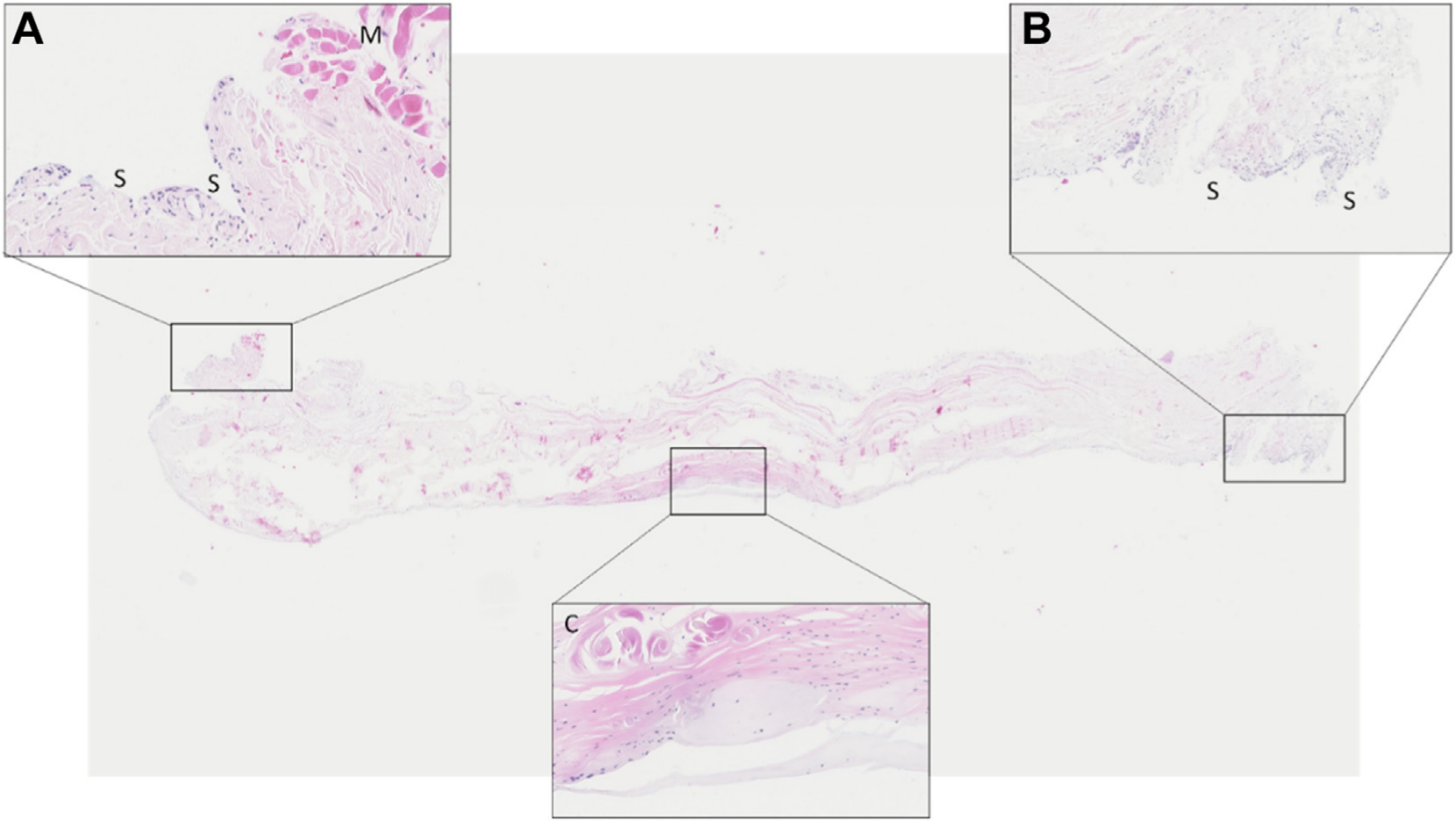
ANL 1; Inset A *Left* (20x): muscle (M) and synovial lining (S) (10x). Inset B *Right* (10x): synovial lining. Inset C central (25x): chondrofication. Chondrofication is present in the *Center* part, on the articulating side opposite of the radial head, of the ANL. The synovial lining is not present in the *Center* part of the ANL. The synovial lining is only present on the lateral parts of the ANL where it attaches to the radial notch of the ulna. Fibrous tissue is present throughout the whole ANL. *ANL*, annular ligament.

**Figure 4:**
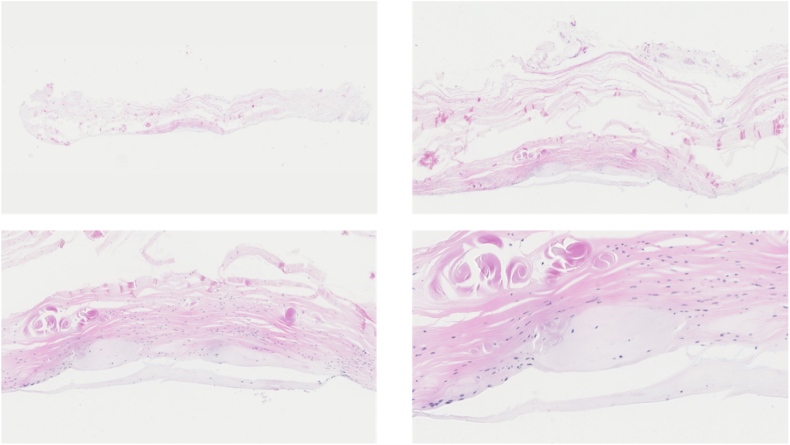
ANL 1; Chondrofication present in the *Center* part, on the articulating side opposite of the radial head, of the ANL. *ANL*, annular ligament.

**Figure 5:**
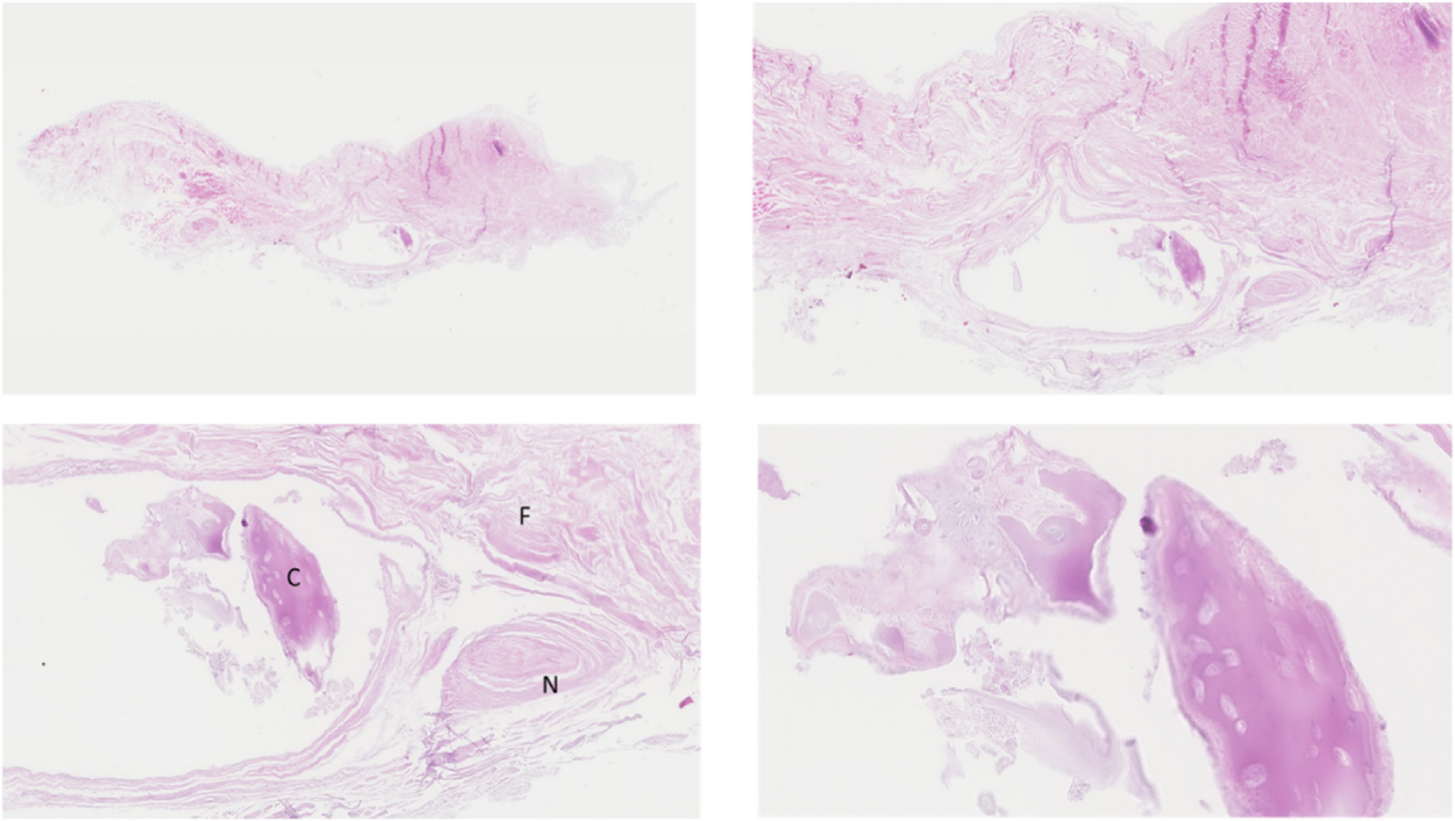
ANL 2; fibrous tissue (F), cartilage tissue (C) and nerve (N). Cartilage tissue with loss of nuclear hematoxylin staining (due to postmortem changes) is present in the *Center* part, on the articulating side opposite of the radial head, of the ANL. No cartilage tissue is present on the lateral sides of the ANL. *ANL*, annular ligament.

**Figure 6:**
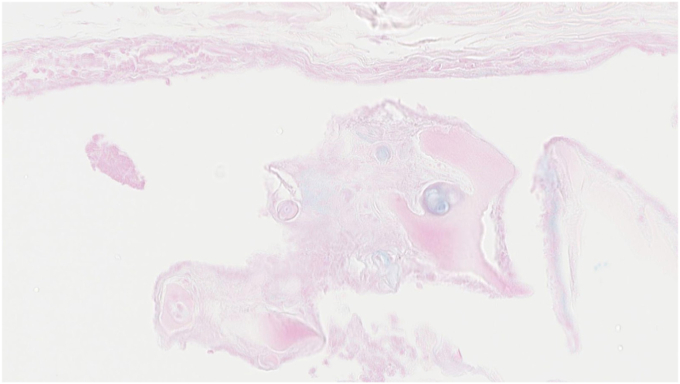
ANL 2; staining Alcian Blue, visualizing the presence of cartilage. *ANL*, annular ligament.

Three ANLs were harvested and examined histologically (Table VII). The PHMS were derived of elderly male (2) and female donors (1). Two samples were from left arms and 1 from a right arm. In one of the ANLs cartilage cells were present. In another ANL chondrofication of fibrinous tissue was found. The ANL showed different types of histological composition in various zones. Interestingly, the cartilage cells and chondrofication were present in the center part, where the ANL was compressed against and experienced friction from the RH. More laterally, only fibrous tissue was observed. In the third ligament no (fibro)cartilaginous tissue was identified. In all ligaments a synovial lining was present. This synovial lining was observed on the lateral parts of the ANL where it attached to the radial notch of the ulna. The synovial lining was absent in the center part of the ANL. Synovial fluid was present between the ANL and RH (Figure 3, Figure 4, Figure 5, Figure 6).

**Table VII:** Overview of the histological examination of 3 ANLs in postmortem human specimens.

Specimen	Gender	Age (yr)	Side	Histology	Synovial lining
ANL 1	Female	87	Left	Chondrofication is present in the center part, on the articulating side opposite of the radial head, of the ANL Fibrous tissue is present throughout the whole ANL	The synovial lining is only present on the lateral parts of the ANL where it attaches to the radial notch of the ulna.
ANL 2	Male	-	Left	Cartilage tissue with loss of nuclear hematoxylin staining (due to postmortem changes) is present in the center part, on the articulating side opposite of the radial head, of the ANL. No cartilage tissue is present on the lateral sides of the ANL.	The synovial lining is only present on the lateral parts of the ANL where it attaches to the radial notch of the ulna.
ANL 3	Male	81	Right	No (fibro)cartilaginous tissue was found	The synovial lining is only present on the lateral parts of the ANL where it attaches to the radial notch of the ulna.

## Discussion

### Overview

This study demonstrated that the findings regarding the histology and tissue composition of the ANL were inconsistent across literature. Our histological examination showed fibrocartilage cells and chondrofication, supporting the results found in some of the previously mentioned articles.^1^^,^^4^^,^^17^ Based on the evidence of the original histological examination and the articles found through the literature search, the authors state that the ANL is a wrap-around ligament that contains (fibro)cartilaginous tissue.

### Fibrocartilage

To date, only 7 articles in medical literature have addressed the tissue composition or histology of the ANL. Two articles^1^^,^^4^ reported the presence of fibrocartilage within the ANL. Anand et al.^1^ did not specify the location of the fibrocartilage cells. However, Bartz et al.^4^ clearly illustrated that in the center part, cartilage cells were evident on its inner, articulating surface. Four of the included articles did perform a histological examination of the ANL but did not identify or report any cartilage cells or cartilaginous tissue in the ANL.^12^^,^^15^^,^^23^^,^^29^ To resolve the contradictions found in literature, the authors performed an original histological study. Three ANL's were dissected and examined. Cartilage cells and chondrofication were present in the center part, on the inner, articulating side of the ANL, where the ligament is compressed against RH.

### Synovial lining

Two articles described the presence of synovial lining on the articulating side of the ANL.^23^^,^^29^ Typically, synovial lining is found on the inner surface of a joint capsule but not on the articulating surfaces. It lacks the ability to withstand compressive forces within the joint. Thus, its presence on the articulating side of the ANL contradicts the hypothesis of a radial-annular joint.^25^ However, Martin et al.^17^ specified that the synovial lining is only present at the distal part of the ANL, extending to the neck of the radius but not covering the articulating surface. In the histological examination done by the authors (JD), a synovial lining was found on all 3 ligaments on the lateral sides of the ANL, near its attachment to the ulna, but not on the articulating part of the ANL.

### Fiber arrangements

Lühmann et al.^15^ expounded on the various types of collagenous fiber arrangements in the elbow joint. The densely packed parallel fiber arrangements in the ANL reflect the stabilizing function of the ANL, corresponding with its function as a wrap-around ligament.

### Definition of a joint

It is important to note that the articulating surfaces between the RH and the ANL cannot be described as a true joint. Joint types are typically classified into 3 types, based on their histological features: fibrous, cartilaginous, and synovial joints. A fibrous joint connects 2 bones with fibrous connective tissue. Cartilaginous joints connect bones by hyaline- or fibrocartilage. Synovial joints are characterized by the ability to move more freely than the other types of joints. The joint cavity filled with synovial fluid prevents friction between 2 articulating bones. In conclusion, a joint is a connection between bones.^14^ Therefore, the articulating surfaces between the ANL and the RH should not be described as a true joint since there is no bone present in the ligament.

### Clinical importance

Although the ANL and RH should not be described as a true joint, the evidence highlights the similarities between a true joint and a wrap-around ligament. This is important for surgical interventions in the elbow. In RH surgery ORIF, plate-and-screw fixation or screw fixation, is a common treatment for partial articular RH fractures blocking motion and complete articular RH fractures that can be sufficiently stabilized to allow for early motion.^22^ While the choice of fixation ultimately depends on the fracture pattern, osteosynthesis material should never be placed within a joint. Plate-and-screw fixation is associated with higher odds of complications compared to buried screw fixation.^32^ The ANL is a wrap-around ligament closely articulating with the RH. Therefore, the authors suggest that in RH surgery buried headless compression screws should be preferred compared to plate-and-screw fixation.^21^^,^^28^^,^^30^

### Limitations

The quality assessment indicated that most included articles did not meet the threshold for ‘good’ or ‘excellent’ quality. Even if an article was rated ‘excellent’ based on the custom-developed scoring system, it would still rank low according to the 2011 Oxford Centre for Evidence-Based Medicine levels of evidence.^9^ However, as this is a scoping review aiming to map the literature, including all available (poor) articles, a knowledge gap is identified showing what future research should focus on. Moreover, the quality assessment tool was not a standardized instrument but a custom-developed system with inherent risk of bias. In addition, most included articles were published more than ten years ago. Advances in techniques and anatomical knowledge in recent years may limit the applicability of these older findings. Therefore, the results of the older articles might be less representative. In addition, Martin et al.^17^ described synovial lining at the distal part of the ANL, but their findings were based solely on anatomical dissection rather than histological examination. The reliability of macroscopic observations regarding synovial lining could be questioned in comparison to histological examinations.

Furthermore, no definitive explanation was found for the discrepancies observed in the histological examinations conducted on the 3 ligaments. These differences might be attributed to variations in age, sex, or lifetime mechanical loading. The specimens were taken from body donors, with a mean age (of the known specimen) of 84 years at the time of death. This limits the generalizability of these findings to the broader population. In addition, the small sample size (n = 3) must be considered when interpreting the results. However, the histological examination was executed thoroughly by cutting up the whole ANL and assessing the material microscopically every 100 μm.

### Conclusion

The ANL is a wrap-around ligament that contains (fibro)cartilaginous tissue. There is no synovial lining present on the articulating surface of the ANL between the ANL and the RH. A wrap-around ligament, closely articulating with the surface of a bone, does not differ from an official joint in terms of function and movement.

## Disclaimers

Funding: No funding was disclosed by the authors.

Conflicts of interest: The authors, their immediate families, and any research foundation with which they are affiliated have not received any financial payments or other benefits from any commercial entity related to the subject of this article.

## Declaration of generative AI and AI-assisted technologies in the writing process

During the preparation of this work the authors used ChatGPT in order to improve the language and readability. After using this tool/service, the authors reviewed and edited the content as needed and take full responsibility for the content of the publication.
